# Books Reviewed

**Published:** 1862-06

**Authors:** 


### BOOKS REVIEWED.

*On Bandaging and other Operations of Minor Surgery; by* F. W. SARGENT, M. D., *member of the College of Physicians of Philadelphia; one of the Consulting Surgeons to Wells' Hospital; new edition, with an additional chapter on Military Surgery, by* W. F. ATLEE, M. D., *and one hnndred and eighty-seven illustrations. Philadelphia:* LEA & BLANCHARD, 1862.

This book presents the Art of Bandaging, and a great many other arts, as they should be practiced, and as they are practiced by all good surgeons. It treats of the Simple Bandage; Compound Bandage; Regional Bandage; Bandages of the Trunk; Bandages for the Upper Extremities; Bandages for the Lower Extremities; Apparatus employed in the treatment of Fractures, Dislocations, and the mechanical means employed in their treatment.

The minor surgical operations are also very carefully and correctly described. Chapters are devoted to the subjects of Rubefacients, Vesicants, Counter Irritants, &c., &c. Methods of Arresting Hæmorrhage; Dressing Wounds; Administering Injections; Introducing Catheter; removing foreign bodies from the passages and canals; diminishing pain during operations, and other kindred processes are fully described.

In addition to all this, the subject of Gun-shot Wounds has been introduced, together with the more important peculiarities in the practice of Military Surgery. The author does not claim originality in the composition of the book, but but has availed himself of the ideas and opinions of others, clothing them in his own style, and giving credit to others, where credit is due. It is especially the "little things" our author would teach us, all the more important since they are so indispensable in surgery, and are often overlooked or neglected in systematic courses of lectures or published treatises on the science.

We have carefully perused this book for the double purpose of what we could learn by so doing, and of finding what was worthy of most especial mention in making our review. The instructions given upon the subject of *Bandaging,* is alone of great value, and while the author modestly proposes to instruct the students of medicine, and the younger physicians, we will say that experienced physicians will obtain many exceedingly valuable suggestions by its perusal.

The description of instruments used in surgical operations; of splints for fractures and mechanical support in dislocations, is worthy of attention, and should be noticed as one of the excellencies of this book.

Without attempting to particularize further, we will conclude our brief notice by saying, that it will be found one of the most satisfactory manuals for reference in the field, or hospital, yet published; thoroughly adapted to the wants of Military surgeons, and at the same time equally useful for ready and convenient reference by surgeons everywhere.

For sale in Buffalo by Breed, Butler & Co. Price $1.75.

---

*Researches and Observations on Pelvic Hæmatocele, by* J. BRYNE, M. D., M. R. C. S. E., *Resident Fellow of the New York Academy of Medicine, &c. New York:* WILLIAM WOOD, 61 *Walker street,* (*late* S. S. & W. WOOD,) 1862.

This is the copy of an article on Pelvic Hæmatocele which has recently appeared in the *Philadelphia Medical and Surgical Reporter*, and part of which constituted a paper read before the New York Academy of Medicine. Of the history of this disease we extract the following: "Without stopping, therefore, to analyze the observations of Ruysch or his contemporaries, it may suffice to remark that *about thirty years* ago, Prof. Racamier, on making incision into the posterior vaginal wall, for the purpose of evacuating the contents of a supposed abscess, discovered that instead of pus, a copious discharge of black, disorganized blood followed. Some years subsequently M. Velpeau, and perhaps one or two others, reported cases of a similar character; but up to 1850, our knowledge of the pathological lesions preceding and accompanying this peculiar kind of tumor, was very limited. The escape of blood into the recto-uterine cul-de-sac of the peritonæum is a fact that has been so long, and often, clearly demonstrated as to leave no room for doubt; but I think it is safe to assert, that up to twelve years ago, the annals of medicine and surgery did not present more than half a dozen well authenticated cases of encysted pelvic hæmatocele. Indeed, as regards British and American medical literature, we might have looked in vain for the slightest allusion to its existence; and even yet, some of our standard works on the diseases of females deny the subject a single chapter."

Our author proceeds to give us the following definition: "The tumor to which the term hæmatocele, or hæmatoma, has been correctly applied, may be defined *an extravasation of blood into or beneath the pelvic peritonæum;* and on account of the space in which this tumor has been, for the most part noticed, it has generally been described as "recto-uterine," "retro-uterine," or "peri-uterine." *But as the extravasated fluid does not invariably select either of the locations thus indicated,* the more correct, and at the same time comprehensive term, of *Pelvic Hæmatocele* should, I think, be used as it includes every form of this affection, whether intra or sub-peritonæal, diffused or encysted."

We are told that this disease most frequently occurs between 25 and 35 years of age, "when the sexual system is in its greatest vigor." "It has also been noticed that the period of invasion is immediately before, during, or soon after the catamenial flow." We then have the predisposing and exciting *causes and symptoms*, and this important topic in the paper under consideration, is very fully and thoroughly considered, and shows the greatest care in research, and the most careful observation. Every physician will be interested and instructed by it.

Great care is also bestowed upon the paragraph devoted to Diagnosis. The difficulty of positive diagnosis is shown, and cases reported where it had been regarded as fibrous tumor, of the posterior uterine walls and encephaloid. Pelvic abscess, retroversion of the uterus, dislocated ovarian cysts, or fibrous tumors, and extra uterine fœtation, are the diseases with which it is most liable to be confounded.

*Treatment*—"Should be three-fold: *Preventive, paliative, curative.*"

Paliative treatment would consist in restoring the equilibrium of the circulation, combating peritonæal complications by local depletion, and other antiphlogistic measures, relieving pain by anodynes, and supporting the patient by proper nourishment.

*Curative treatment* consists in operations upon the tumor, though it has been claimed by some surgeons that we "can always rely on absorption." It has been freely opened, punctured; contents drawn with trocar, such as is used for ovarian cysts. This last is the course advised and favored by our author, and his conclusions seem altogether sustained by the history of the cases which have been introduced.

We must not attempt to follow further, though his paper is of the highest importance, and has interested and instructed us very much. It shows great labor of research, much careful observation, guided by an intelligent experience, and will be read with interest and profit. It is received in pamphlet form of 44 pages, and we hope those who have not already seen it, will do so without delay.

---

HEALTH; *Five Lay Sermons to Working People by* JOHN BROWN, M. D., *Author of* "*Rab and his Friends,*" *etc. New York:* ROBT. CARTER & BRO'S, *No* 530 *Broadway*, 1862.

John Brown has said some very good things to the working people in his five sermons. The text of the first is "The Doctor—our duties to him."—

He says, "Therefore I shall ask you to remember *four things* about your duty to the doctor, so as to get the most good out of him, and do the most good to him.

1st—It is your duty to trust the doctor.

2d—It is your duty to obey the doctor.

3d—It is your duty to speak the truth to the doctor, the whole truth, and nothing but the truth, and

4th—It is your duty to *reward* the doctor."

By way of closing the first sermon he gives the working people the following warning, a warning which would perhaps be of equal service to the ladies and gentlemen of leisure.

"One person I would earnestly warn you against, and that is, the *Quack Doctor*. If the real doctor is a sort of god of healing, or rather our God's cobbler for the body, the quack is the devil for the body; and like his father, he is a great liar and cheat. He offers you what he cannot give. Whenever you see a medicine that cures everything, be sure it cures nothing; and remember, it may kill. The devil promised our Saviour all the kingdoms of the earth, if he would fall down and worship him; now this was a lie, he could not give him any such thing. Neither can the quack give you his kingdoms of health even though you worship him as the best likes, by paying him for his trash. He is dangerous, dear, and often deadly; have nothing to do with him."

We cannot follow and give any account of what is said in the four remaining sermons, but suffice it to say, they contain much valuable instruction for working people and all others.

